# Supplementary figures and images for: Preservation of microscopic fur, feather, and bast fibers in the Mesolithic ochre grave of Majoonsuo, Eastern Finland
Source: PLoS One. 2022 Sep 27;17(9):e0274849. doi: 10.1371/journal.pone.0274849 (PMC9514644; doi:10.1371/journal.pone.0274849)

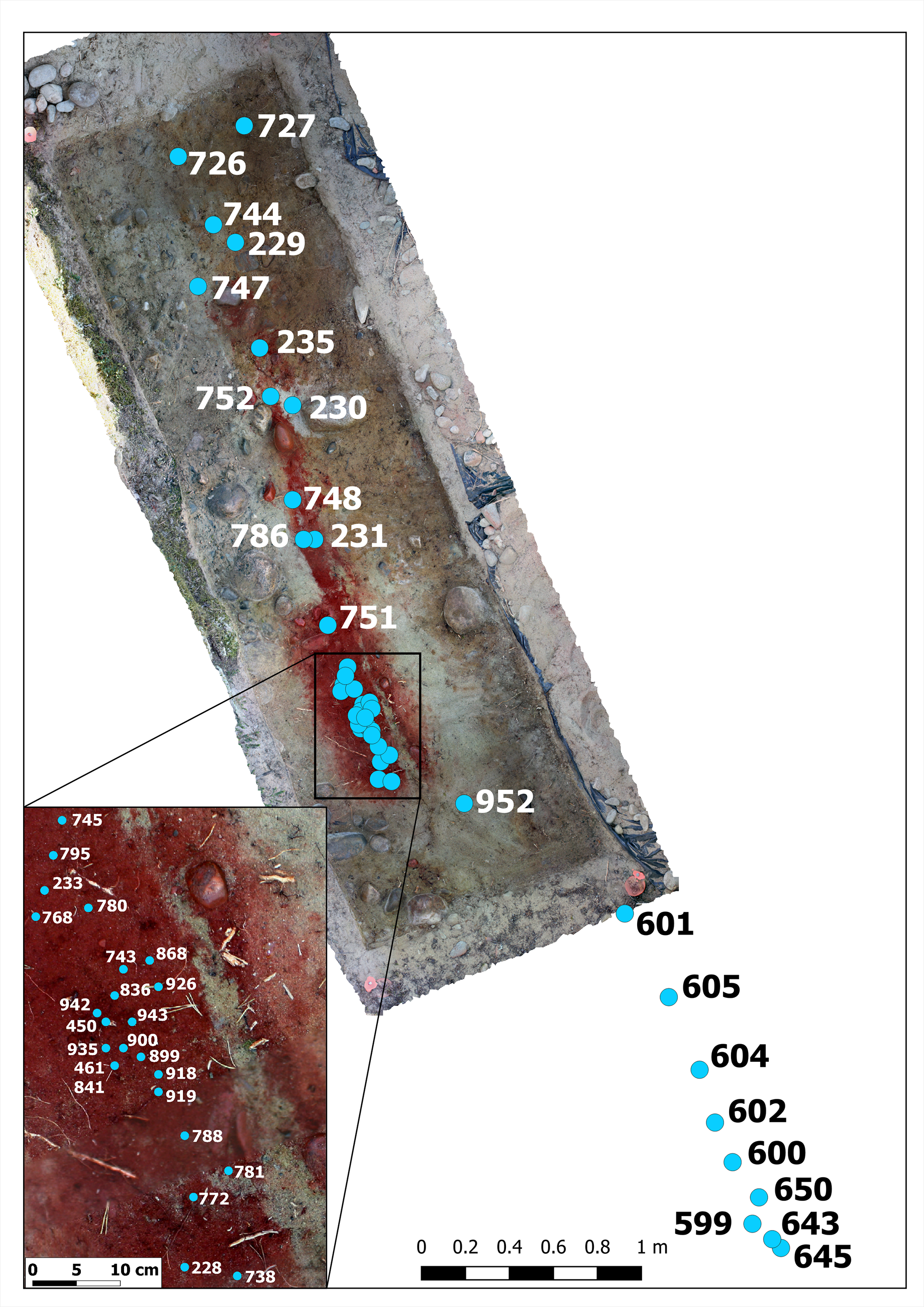

Supplement: S1 Appendix — Background map: Orthophoto of the photogrammetry 3D model, made by Jan-Erik Nyman, Esa Mikkola and Janne Rantanen, Finnish Heritage Agency. Drawing: Johanna Roiha. (TIF) [file pone.0274849.s003.tif]
