# Supplementary material for: Preservation of microscopic fur, feather, and bast fibers in the Mesolithic ochre grave of Majoonsuo, Eastern Finland
Source: PLoS One. 2022 Sep 27;17(9):e0274849. doi: 10.1371/journal.pone.0274849 (PMC9514644; doi:10.1371/journal.pone.0274849)
Supplement: S2 Appendix — (DOCX) [file pone.0274849.s004.docx]

Table 1. Descriptive statistics of colour Cielab parameters in the three groups described and the total of all samples.

|  | L* | a* | b* | C* | hue |
| --- | --- | --- | --- | --- | --- |
| Total | 66,3 ± 8.3 | 8,3 ± 7,6 | 14,8 ± 6.6 | 17,3 ± 9.4 | 67,8 ± 13.4 |
| Group-1, no red | 72,1 ± 6.1 | 1,4 ± 0.8 | 8,3 ± 2.1 | 8,4 ± 2.2 | 81,1 ± 4.1 |
| Group-2, low red | 66,9 ± 3.8 | 8,6 ± 2.7 | 16,6 ± 1.8 | 18,7 ± 2.8 | 63,1 ± 5.2 |
| Group-3, red | 55,9 ± 4.5 | 19,2 ± 2.7 | 23,5 ± 1.3 | 30,4 ± 2.7 | 50,9 ± 2.5 |


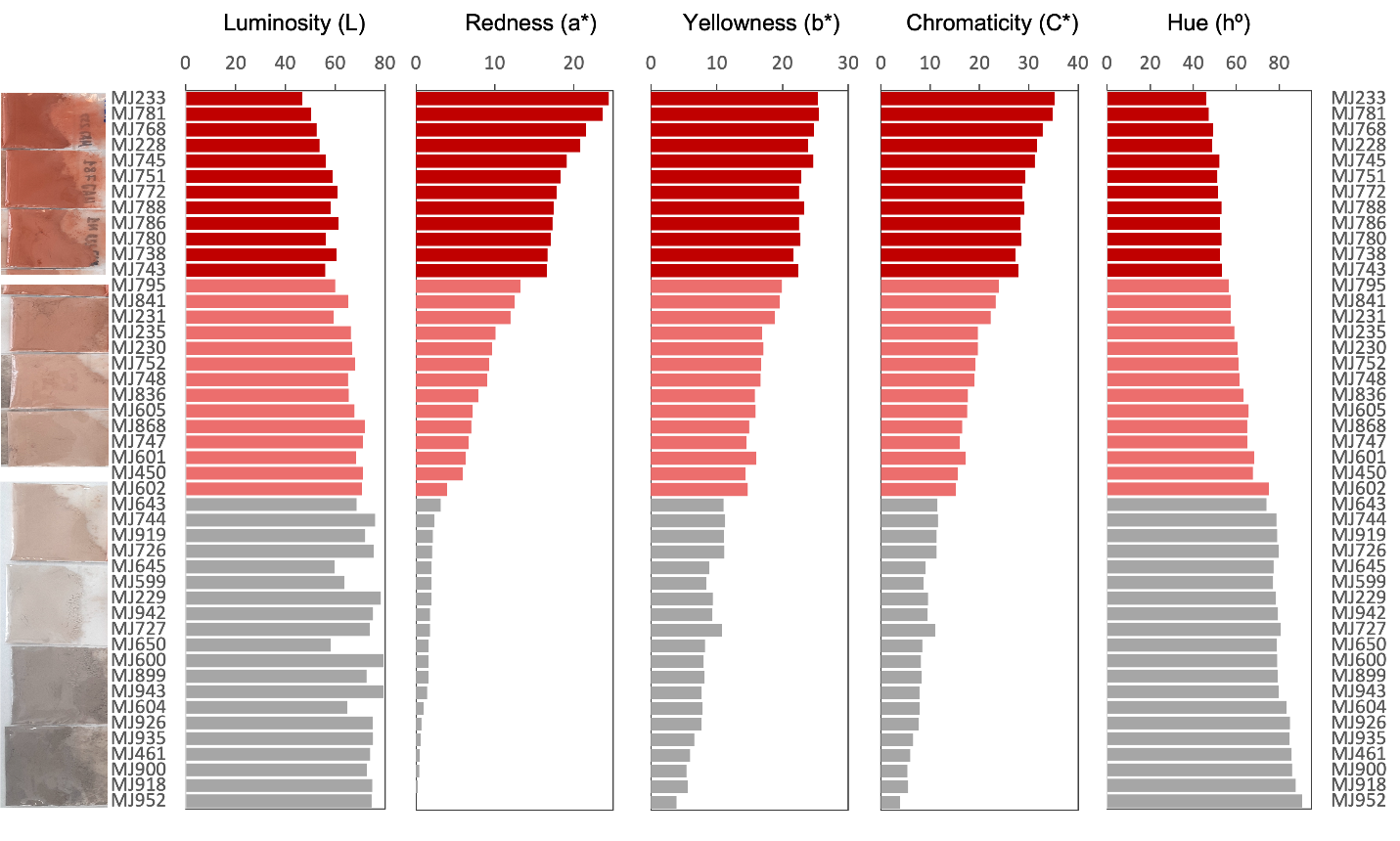
Fig 1. Distribution of colour properties in the analyzed samples. Samples are classified according to redness (a*). Pictures of samples are placed to the left side to see the actual colours.
