# Supplementary material for: Preservation of microscopic fur, feather, and bast fibers in the Mesolithic ochre grave of Majoonsuo, Eastern Finland
Source: PLoS One. 2022 Sep 27;17(9):e0274849. doi: 10.1371/journal.pone.0274849 (PMC9514644; doi:10.1371/journal.pone.0274849)
Supplement: S3 Appendix — (DOCX) [file pone.0274849.s005.docx]

Table 1. Fatty acid composition (mol%) of six soil samples.


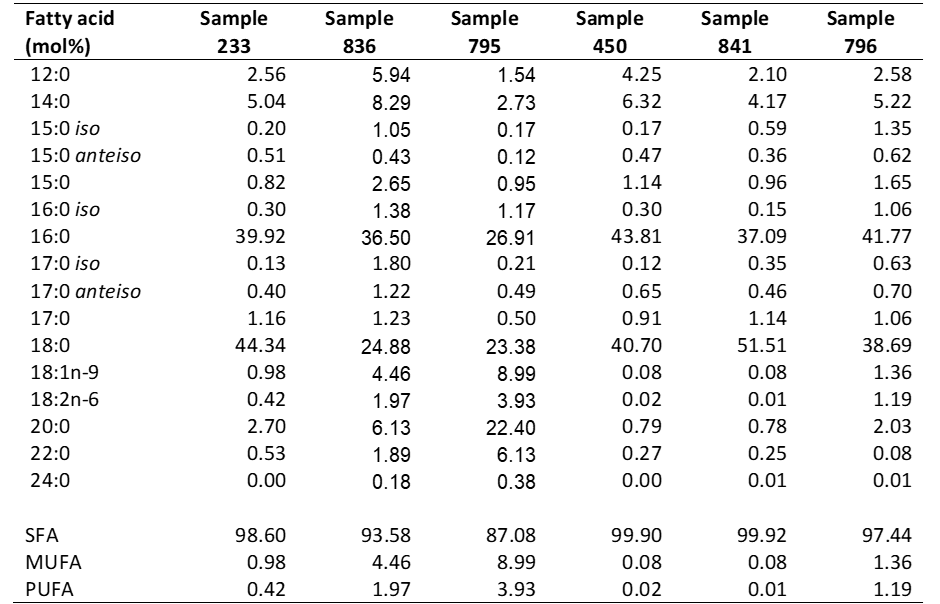


Fatty acids are abbreviated: [carbon number]:[number of double bonds in the chain]n-[position of the first double bond calculated from the methyl end]. The markings *iso* and *anteiso* refer to a branch in the chain that locates in *iso* or *anteiso* position, respectively. SFA, MUFA and PUFA are the sums of the saturated, monounsaturated and polyunsaturated fatty acids, respectively.


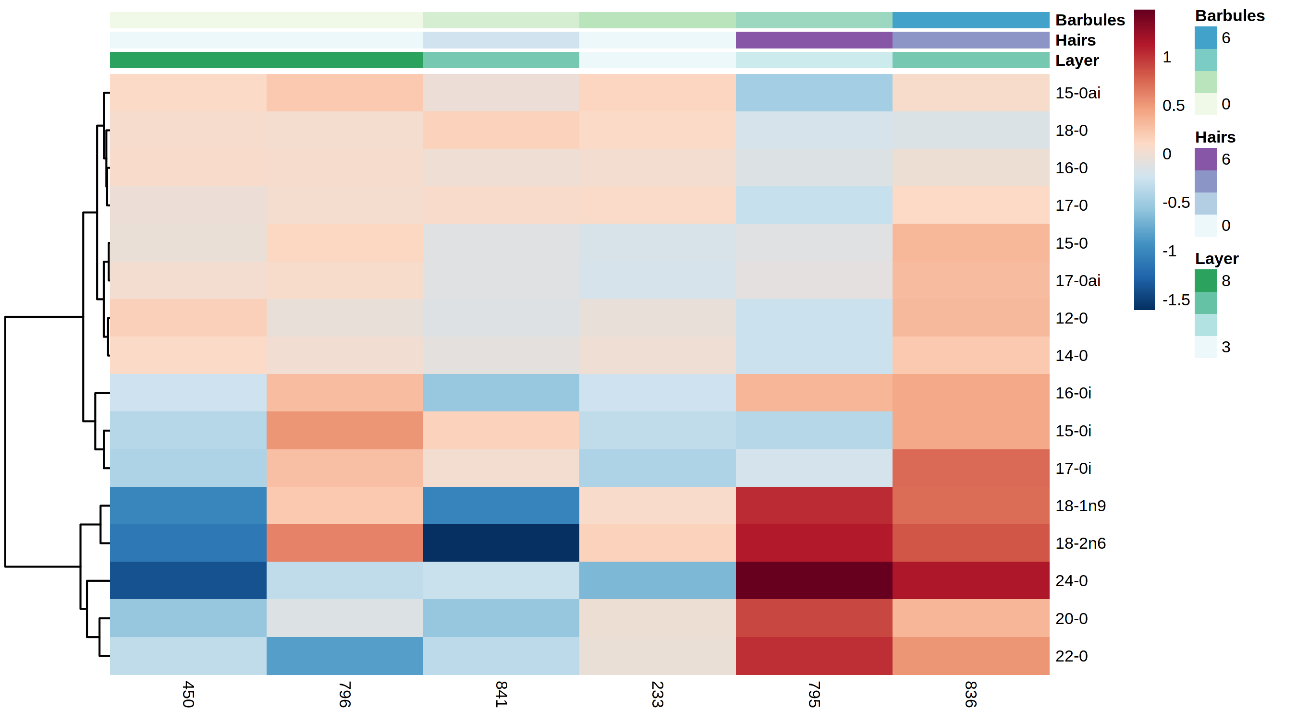


Fig. 1. Heat map illustrating differences in the fatty acid composition among the six soil samples, which were arranged horizontally according to the compositional similarity/difference. The samples 836, and 795 stand out from the others. The number of avian barbules and mammalian hairs found in the samples, and the depth of the sampled soil layer are shown on the uppermost three rows of the map. Fatty acid abbreviations as in Table 1 but in condensed form (: to -; *anteiso* to ai; *iso* to i).
